# Review on Perioperative and Oncological Outcomes of Robotic Gastrectomy for Cancer

**DOI:** 10.3390/jpm11070638

**Published:** 2021-07-06

**Authors:** Giuseppe Giuliani, Francesco Guerra, Lorenzo De Franco, Lucia Salvischiani, Roberto Benigni, Andrea Coratti

**Affiliations:** USL Toscana Sud Est, Misericordia Hospital, 58100 Grosseto, Italy; francesco.guerra@uslsudest.toscana.it (F.G.); lorenzogiacinto.defranco@uslsudest.toscana.it (L.D.F.); lucia.salvischiani@uslsudest.toscana.it (L.S.); roberto.benigni@uslsudest.toscana.it (R.B.); andrea.coratti@uslsudest.toscana.it (A.C.)

**Keywords:** minimally invasive gastrectomy, robotic gastrectomy, laparoscopic gastrectomy, gastric cancer

## Abstract

Background. Minimally invasive gastrectomy is currently considered a valid option to treat gastric cancer and is gaining increasing acceptance. Recent reports have suggested that the application of robots may confer some advantages over conventional laparoscopy, but the role of robotic surgery in clinical practice is still uncertain. We aimed to critically review the relevant evidence comparing robotic to standard laparoscopic surgery in performing radical gastrectomy. Methods. The Pubmed/Medline electronic databases were searched through February 2021. Paper conference and the English language was the only restriction applied to our search strategy. Results. According to the existing data, robotic gastrectomy seems to provide some benefits in terms of blood loss, rate of conversion, procedure-specific postoperative morbidity, and length of hospital stay. Robotic gastrectomy is also associated with a longer duration of surgery and a higher economic burden as compared to its laparoscopic counterpart. No significant differences have been disclosed in terms of long-term survivals, while the number of lymph nodes retrieved with robotic gastrectomy is generally higher than that of laparoscopy. Conclusions. The current literature suggests that robotic radical gastrectomy appears as competent as the conventional laparoscopic procedure and may provide some clinical advantages. However, due to the relative paucity of high-level evidence, it is not possible to draw definitive conclusions.

## 1. Introduction

The employment of laparoscopic techniques in gastric surgery has diffused and evolved rapidly over the last two decades [1,2,3]. Minimally Invasive Gastrectomy (MIG) is now almost universally accepted as a valid option for the treatment of gastric cancer (GC) as it optimizes postoperative recovery without compromising the adequacy of resection and long-term oncological outcomes [1,2,3,4,5,6]. Recent NCCN guidelines (version 2.2021) for GC treatment indicate that MIG (including both conventional laparoscopic and robotic techniques) may be considered, for early and locally advanced cases, provided specific expertise in minimally invasive foregut procedures and lymphadenectomy are available [1,2,3,5,6,7,8,9].

The technical difficulties of laparoscopic surgery that are encountered in performing MIG have led some surgical teams to the use of robotic surgery [10,11]. Indeed, robotics has been originally introduced in clinical practice to overcome some of the intrinsic limitations of conventional laparoscopic and to broaden the range of application of minimally invasive surgery, and also in the field of the surgery of the stomach, several experiences have indicated excellent outcomes [3,5,6,7,8,9]. A recent study by Stewart et al. have investigated the adoption of robotic surgery in surgical oncology by analyzing the available data from the National Inpatient Sample (NIS) American database [12]. The authors showed that over 5 years (2010–2014), there was a 5-fold increase in the application of robotic surgery. Of note, the application of robotic gastrectomy (RG) increased from 2% in 2010 to 6% in 2014. However, robotic surgery of the stomach is still in its early stages and its actual role in clinical practice is still to be defined [13,14]. Current evidence essentially indicates non-inferiority of RG to standard Laparoscopic Gastrectomy (LG), but general conclusions on whether RG provides clear advantages over LG are still difficult to draw, as available data is mainly derived from low-level analyses returning highly variable results [15,16,17,18]. Accordingly, this paper aims to analyze the existing evidence on RG for cancer with special emphasis on comparing the employ of robotic technology to conventional LG.

## 2. Methods

Institutional review board approval and written consent were not needed for this paper. The last version of PRISMA Statement (Ref) checklist for reporting systematic reviews and meta-analysis, was used as guide to aggregate the available scientific papers comparing RG and LG [19].

Two authors (GG, FG) performed an independent literature search up to APRIL 2021. The PubMed/MEDLINE electronic databases were queried with the following search strings: ‘‘robot-assisted surgery’’ and “laparoscopic” and ‘‘gastric cancer’’ or “gastric neoplasm” and ‘‘gastrectomy’’ or ‘‘gastric resection’’. All article typologies were considered eligible except for conference proceedings, case reports, and small series with less than ten patients. Reviews, meta-analyses, and original articles were included in our analysis based on the following features: novelty, caseload, topic, impact, and availability of raw data. Our search criteria were also restricted to the English language. Independently, the two authors screened titles and abstracts of the retrieved records. Full-text versions of the papers deemed suitable for inclusion were appraised, and relative references were screened to identify additional, eligible articles. Differences in opinion were discussed with the input of a third author (DFL).

Suitable studies were thus evaluated and pooled in the review if the following criteria were met:

- inclusion of adult patients undergoing robotic or laparoscopic total or distal gastrectomy for gastric cancer;
- robotic and standard laparoscopic approach comparator intervention;
- data on perioperative, post-operative, and oncological outcomes.

Continuous data are presented as mean ± standard deviation, whereas categorical data are expressed as absolute numbers and percentages.

## 3. Results

**Figure 1:**
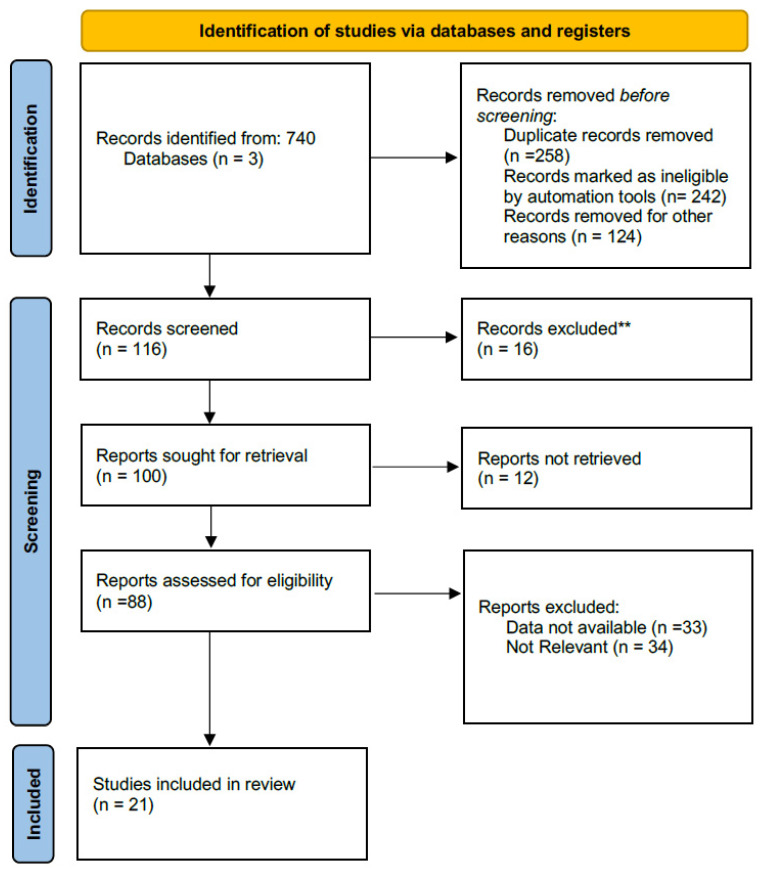
Search Strategy: Page MJ, McKenzie JE, Bossuyt PM, Boutron I, Hoffmann TC, Mulrow CD, et al. The PRISMA. 2020 statement: an updated guideline for reporting systematic reviews. *BMJ* **2021**, *372*, n71, doi:10.1136/bmj.n71.

Our initial electronic search yielded 740 titles. Titles and abstracts were evaluated, and duplicate records were excluded. Full texts with relative bibliographies were thus appraised, and a total of 21 studies were eventually deemed suitable for inclusion and data extraction [4,5,6,8,13,15,17,18,20,21,22,23,24,25,26,27,28,29,30,31,32]. The selection process is given in Figure 1, while Table 1 describes the general characteristics of the included studies. There were 2 randomized controlled trial, 2 prospective study, 4 meta-analysis, 13 retrospective analyses.

### 3.1. Perioperative Details

Regardless of the type of procedure, one of the main limitations of robotic surgery is operative time [12,18,19]. Several studies, including several meta-analyses, indicate that the duration of surgery for RG is invariably higher than for LG, for both total and distal gastrectomy [4,5,12,16,18,19,20]. However, such difference seems to be attenuated by the progress of specific experience with the technique. A recent study by Nakauchi et al., from the Memorial Sloan Kettering Cancer Center, reporting their experience with radical gastrectomy over 10 years. The authors started performing RG after an extensive experience with conventional LG. Interestingly, and in contrast with the majority of previous reports, the analysis of intraoperative outcomes showed that RG had a shorter operative time compared to LG [21].

Published studies comparing RG to LG almost regularly indicate that patients receiving MIG with the robot have reduced perioperative Estimated Blood Loss (EBL). High-level randomized studies mirror this evidence, which is in turn, consistent with recent meta-analyses appraising the issue [12,17,22].

Unplanned conversion into an open procedure has been conventionally utilized to evaluate the proficiency of minimally invasive surgery. A lower conversion rate has been reported as a theoretical advantage of robotic surgery over conventional laparoscopic surgery [33,34]. Emerging data from a recent meta-analysis, including 40 retrospective studies and 17.712 patients, demonstrated no statistical difference in conversion rate between RG and LG. [13]

However, a retrospective study comparing robotic and laparoscopic approaches for radical gastrectomy including 311 MIG, over ten-year experience from the Memorial Sloan Kettering Cancer Center, showed that RG had fewer conversion rate compared to LG (25.8% vs. 39.7%; *p* = 0.010) [21].

Compared to the laparoscopic approach, three-dimensional view, wrist movements and the stable traction of the robotic instruments provide a delicate and fine dissection that might explain the reduction of EBL as well as the trend of a lower conversion rate of RG.

### 3.2. Postoperative Outcomes

Currently, the only available RCTs comparing LG and RG are those published by Pan et al. [23] and Lu et al. [24]. Some 163 patients who underwent MIG with curative intent for AGC were included in the trial published in 2017 by Pan et al. RG was associated with significantly reduced blood losses and a higher number of yielded lymph nodes [23]. As for postoperative outcomes, patients in the RG group experienced significantly less postoperative pain and had an earlier functional recovery, including abbreviated length of hospital stay as compared to the LG group. On the other hand, the rate of postoperative complications favored RG over LG, but this difference did not disclose any statistical significance.

In the more recent trial by Lu et al., the authors compared the outcomes of patients receiving distal gastrectomy by surgeons with large expertise in MIG (>300 LG and 50 RG before trial initiation) [24]. In this trial, a total of 141 and 142 well-balanced patients were assigned to the RG group and the LG group, respectively. While no significant difference in the length of postoperative hospital stay was registered, interestingly, the overall incidence of postoperative morbidity was significantly lower for RG as compared to LG, mostly in terms of medical complications. Multivariate analysis demonstrated that RG was an independent protective factor for postoperative complications. The RG and LG groups did not differ significantly on the number of examined lymph nodes. However, the number of extra-perigastric stations (7–9, 11p and 12 a) was significantly higher following RG than LG, and the percentages of procedures resulting in lymph node noncompliance (defined as the absence of lymph nodes from more than one lymph node required station) was significantly lower for RG than LG (24.8% vs. 40.1%), mainly owing to different results for the extraperigastric regions.

A growing number of further, nonrandomized recent reports have suggested potential advantages of RG over LG in terms of postoperative complications [7,24,25,26,27]. Interestingly, such benefits seem to be enhanced in the setting of more technically demanding procedures, such as total and proximal MIG, completion of total gastrectomy for remnant gastric cancer, and MIG in patients who are obese or who present with advanced disease [5,13,26,28,29,30].

Kinoshita et al. very recently, published the results of an interesting retrospective, single-institutional, case-controlled study including nearly 1200 patients receiving MIG with either a robotic or conventional laparoscopic approach [26]. All included patients had primary, stage I-III gastric malignancy. All types of MIG were considered in the analysis, including a relatively high percentage of total gastrectomy (15–26%) and proximal gastrectomy (11–15%). Overall, although the two groups were well-matched in terms of general baseline characteristics, in the RG group there were more patients with advanced disease (clinical stage III) and there was a higher proportion of patients receiving total or proximal gastrectomy as compared to the LG group. Despite this, patients in the RG group had a significantly lower incidence of postoperative Clavien-Dindo grade II and III complications than those in the LG group [35]. A multivariate analysis of factors affecting postoperative morbidity, a robotic approach was significantly associated with reduced incidence of complications.

Alhossaini and colleagues retrospectively reviewed the outcomes of 55 patients who received minimally invasive completion gastrectomy for malignancy arising in the gastric remnant [28]. The authors compared 30 patients who had surgery with conventional laparoscopy with those whose procedure was accomplished with the robot. Overall, no significant differences were noted between the two groups of patients, neither in terms of perioperative data nor in terms of postoperative morbidity. Of note, though this data did not reach statistical significance (*p* = 0.058), there was a 13% rate of cases of unplanned conversion into an open procedure in the LG, while all RGs were completed in a minimally invasive technique.

A recent article by Wang et al. assessed the severity and rate of incidence of postoperative complications according to the Clavien—Dindo classification system following RG and LG for AGC [29]. The authors conducted a retrospective analysis of >500 MIGs which were matched between RG and LG with the propensity score matching method. RG was associated with a larger amount of examined lymph nodes as compared to LG (mean 40.8 vs. 37.1), while no differences were found in terms of perioperative blood loss and, of note, duration of surgery. Interestingly, they found that the incidence of overall and severe (defined as grade IIIa or greater events) morbidity following RG was significantly reduced as compared to LG (24.5% vs. 18.8%, and 8.9% vs. 17.5%, respectively, *p* = 0.002). This also translated into a shorter postoperative hospitalization for RG as compared to LG. In addition, at univariate analysis, together with age, BMI, advanced disease, duration of surgery, and total gastrectomy, LG was significantly associated with a higher risk of overall and severe morbidity.

### 3.3. Oncological Outcomes

Concerning long-term oncological outcomes, only limited data is still available [5,6,13,15,17,27]. One of the most robust data currently available is that by Shin et al., who recently published the results of a propensity score-weighted analysis of more than 2000 patients with GC receiving MIG (either LG or RG) with curative intent [5]. After patients matching, no differences were noted on the total number of harvested lymph nodes, while that of supra-pancreatic lymph nodes were higher in the RG. Interestingly, in terms of oncological findings, there was no statistically significant difference between LG and RG neither on OSs nor in DFSs at both unweighted and weighted analyses.

Analog findings have been indicated by Li et al., who reported on the long-term oncological outcomes of RG vs. LG in the treatment of patients with locally advanced GC [27]. Their study included nearly 1200 patients whose outcomes were compared between RG and LG using one-to-one propensity score matching. The 3-year DFS rates favoured RG (76%) over LG (70%) without statistical significance (*p* = 0.07). As for 3-year OS, there was a nonsignificant benefit following RG (77%) as compared to LG (73%).

Some of reviews have also investigated potential differences between RG and conventional LG, without identifying any significant advantage of one technique over the other [13,14,17]. The latest meta-analysis comparing the oncological outcomes of RG vs. LG has been published by Yang et al. and colleagues and combines the data reported in 11 articles including a total of nearly 4100 patients [17]. Overall, there was a nonsignificant OR of 0.98 and 0.53 favoring RG over LG in terms of 3-year and 5-year OS, respectively. Similarly, data on recurrences indicated that the two groups of treatment did not differ significantly (OR = 0.88).

It is well-known that the number of harvested lymph nodes and surgical margins are currently considered as most appropriate indicators of oncologically adequate resections [13]. Most comparisons between RG and LG, indicate that the median amount of lymph nodes harvested is higher during RG than LG [18,21,24,25,30]. The currently two available randomized trials upon the argument, as already mentioned, support an advantage of RG over LG on lymphadenectomy [23,24]. Other observational reports and several recent meta-analyses echo such findings [8,13,14,25,30], also in obese patients. Choi et al. recently published a study that compared short and long terms outcomes of open, laparoscopic, and robotic radical gastrectomy with D2 lymphadenectomy in obese patients. Among 185 patients with a median BMI of 26.5 kg/m^2^, there were 69 open, 62 laparoscopic, and 54 robotic procedures. RG resulted in a greater mean number of retrieved lymph nodes, and a higher rate of lymph nodes harvest compliance compared to LG and OG.

With the purpose to evaluate the impact of MIS on survival in the United States, Hendriksen et al. analyzed the data of >17,000 patients who underwent gastrectomy for adenocarcinoma between 2010 and 2015 [6]. Overall, after propensity score matching, MIG resulted in a significantly improved 5-year survival compared to open surgery. Interestingly, while no difference was disclosed for long-term survival between patients receiving LG vs. RG, the percentage of patients receiving adequate lymph nodes sampling was significantly higher for RG (60%) as compared to LG (51%).

However, the most recent publication by Guerrini et al. represents the largest meta-analysis currently available and combines the results of 40 retrospective studies including nearly 18,000 patients receiving either laparoscopic or robotic MIG [13]. Concerning oncological outcomes, RG demonstrated a significantly increased mean number of yielded lymph nodes as compared to LG.

### 3.4. Costs

In general, the economic burden of robot-assisted surgery is substantially higher than that of conventional laparoscopic surgery and previous reports consistently indicate that costs associated with RG are higher as compared to standard laparoscopic MIG [13,14,15,18,32]. In the above-mentioned RCT by Lu et al. a specific analysis of costs was reported. The increased cost was found in RG as compared to LG in terms of total and indirect costs [24]. Interestingly, the direct cost of RG was significantly lower than that of LG, with specific economic advantages on surgical procedures, hospitalization, laboratory and radiology tests, and perioperative transfusions.

Actually, robotic surgery is evolving rapidly, and probably a reliable evaluation of its actual economic impact on clinical practice should include some indirect aspects, most of which are not fully predictable at present [36,37]. The effect due to possible differences between RG and LG on postoperative morbidity, length of hospitalization, and incidence of unplanned conversion should be considered over time [34,36,37]. In addition, given the exponential evolution of robotic surgery, it is likely that different robotic systems will enter the market in the near future resulting in more competition and lower purchase and maintenance costs of robotic platforms [34,36,37,38].

## 4. Discussion

The studies included in our updates on robotic gastrectomy for cancer demonstrate superior surgical outcomes of RG as compared to LG, especially in terms of perioperative blood losses, rates of conversion, procedure-related postoperative morbidity, and length of hospital stay. On the other hand, the duration of surgery and costs associated with the application of robotic surgery are consistently higher than those of LG [23,24,25,28,30]. Finally, RG is generally associated with a greater lymph node yield, while long-term outcomes seem to be substantially comparable [5,13,14,17].

Operative time is considered one of the limitations of the robotic approach: several studies demonstrated a longer operative time for RG than for LG [4,5,13,17,20]. However, such difference seems to be attenuated by the progress of specific experience with the technique. Recently Nakauchi et al. reported their experience with radical gastrectomy over 10 years from the Memorial Sloan Kettering Cancer Center. The authors compared OG with MIG and RG with LG. Interestingly, and in contrast with the majority of previous reports, the analysis of intraoperative outcomes showed that RG had a shorter operative time compared to LG (212 vs. 240 min *p* < 0.001) [21]. They concluded that this finding may reflect their institutional experience because the authors started performing RG after an extensive experience with conventional LG (more than 100 LG).

A lower conversion rate has been reported as a theoretical advantage of robotic surgery over conventional laparoscopic surgery [33,34].

Conversion negatively impacts postoperative outcomes, may postpone the initiation of adjuvant treatments, and ultimately translate into poorer long-term survivals [33,34,39].

We recently conducted a meta-analysis of available randomized evidence comparing robotic and conventional laparoscopic surgery to identify possible differences in the rate of unplanned conversion to open surgery [34]. From twelve trials, 1867 patients undergoing surgery with curative intent for abdominal malignancy were included in the analysis. The study included 443 patients treated for gastric cancer, both distal and total gastrectomy. Overall, the rate of conversion was 4.8%, 3.1%, and 6.5% being the relative incidence for robotic and laparoscopic surgery, respectively. At meta-analysis, this difference was significant, with an OR of 0.56 favoring robotic over laparoscopic surgery (*p* = 0.03).

Robotic gastrectomy compared to the laparoscopic conventional approach is associated with a lower incidence of postoperative complications. In the RCT published by Pan et al. in 2017, this difference did not reach statistical significance [23]. Nevertheless, in the more recent trial by Lu et al., in which a total of 141 and 142 well-balanced patients were assigned to the RG group and the LG group, respectively the overall incidence of postoperative morbidity was significantly lower for RG as compared to LG, mostly in terms of medical complications. Furthermore, at multivariate analysis the authors demonstrated that RG was an independent protective factor for postoperative complications [24].

However, these findings seem to be enhanced in the setting of more technically demanding procedures, such as total and proximal gastrectomy, completion total gastrectomy for remnant gastric cancer, as well as in patients who are obese or who present with advanced disease [5,13,26,27,28].

Pancreatic morbidity deserves a specific consideration when specific post-operative complications after MIG are analyzed. Pancreatic injury due to intraoperative manipulation during dissection and specifically lymphadenectomy may lead to acute pancreatitis, associated or not with pancreatic fistula formation [40,41,42]. The real incidence of such events is probably underestimated in clinical practice and likely difficult to ascertain. Still, pancreas-related morbidity following gastrectomy should be taken into consideration in the assessment of radical MIG, owing to the potential to significantly aggravate the postoperative course of patients [40,41,42]. The available evidence on the incidence of pancreas-related complications following radical MIG was investigated in a specific meta-analysis conducted by our group and published in 2018 [41]. RG was compared with LG on the relative incidence of postoperative acute pancreatitis and postoperative pancreatic fistula (POPF). A slight advantage of RG over LG was disclosed, albeit with marginal statistical significance. By analyzing more than 2000 patients from 8 comparative studies, the incidence of pancreatic events following MIG was 2.2%, ranging from 0.8% to 16.2%. Despite patients in the LG tended to have higher BMI and more advanced disease, 1.7%, and 2.5% were the relative rates of pancreas-related morbidity for RG and LG, respectively. In particular, the incidence of POPF favored RG over LG (2.7% vs. 3.8%) with an OR of 0.8, albeit without statistical significance (*p* = 0.69).

Although, long-term outcomes seem to be substantially comparable after RG and LG [17,27], interestingly, Choi et al. comparing long-term oncological outcomes (with a median follow-up of 56 months) of open, laparoscopic, and robotic radical gastrectomy with D2 lymphadenectomy in obese patients, demonstrated that RG compared to OG and LG was a protective factor for recurrence-free survival at multivariable analysis [30].

This finding may reflect the greater mean number of retrieved lymph nodes that seems to be associated with the robotic approach [6,8,13,14,23,24,25,30]. Three-dimensional video system, wrist movements, the stable traction of the robotic instruments as well as ICG fluorescent lymphography may explain the superior lymphadenectomy performance associated with RG [43].

In conclusion, in the last years, there has been probably too much enthusiasm around the introduction of the robot in surgical practice. Actually, despite possible specific merits over conventional LG, the relative lack of high-level evidence still precludes the possibility to reach definitive conclusions [8,13,14,24]. Randomized studies are difficult to run, as most referral centers dedicated to MIG have consolidated experience either in LG or in RG, and different levels of specific expertise are likely to affect monocentric comparisons [29,34]. Actually, both laparoscopic and robotic MIG can be recommended for selected patients at centers with specific technical expertise, as they are associated with fewer morbidity while resulting in long-term outcomes which are equivalent to those of open surgery [3,4,16,21]. Available data from RCT is scarce and most retrospective reports are still biased by some confounding factors. As a consequence, the question of whether RG presents significant advantages over conventional LG for GC remains an avenue for further research.

## Figures and Tables

**Table 1:** General characteristics of the included studies.

Author	Year	Study	Origin	Approach	n. Patients	Type of Resection
Kim HI et al.	2016	Prospective	Korea	LG/RG	434	TG/DG/PPG/PG
Chen K et al.	2017	Mata–Analysis	China	LG/RG	5953	TG/DG/PG
Pan HF et al.	2017	RCT	China	LG/RG	163	TG/DG/PG
Hendriksen BS et al.	2018	Retrospective	USA	LG/RG/OG	17.449	TG/ DG
Liu H et al.	2019	Retrospective	China	LG/RG	20	DG
Wang WJ et al.	2018	Retrospective	China	LG/RG	527	TG/DG
Uyama I et al.	2018	Prospective	Japan	RG	326	TG/DG/PG
Gao, Y Q et al.	2019	Retrospective	China	LG/RG	502	TG/DG/PG
Bobo Z et al.	2019	Mata–Analysis	China	LG/RG	4576	TG/DG
Hikage M et al.	2020	Retrospective	Japan	LG/RG	1208	TG / DG/PPG/PG
Guerrini GP et al.	2020	Mata–Analysis	Italy	LG/RG	17.712	TG/DG/PPG/PG
Yang P. et al.	2020	Mata–Analysis	China	LG/RG	4142	TG/DG
Li ZY et al.	2020	Retrospective	China	LG/RG	1476	DG
Alhossaini RM et al.	2020	Retrospective	Korea	LG/RG	55	CG
Bolger JC et al.	2021	Retrospective	Ireland	LG	258	TG/DG
Shin HJ et al.	2021	Retrospective	Korea	LG / RG	2084	TG/ DG
Nakauchi M et al.	2021	Retrospective	USA	LG/RG/OG	845	TG/DG/PG
Lu J et al.	2021	RCT	China	LG/RG	300	DG
Tian Y et al.	2021	Retrospective	China	LG/RG	1686	TG/DG
Kinoshita T et al.	2021	Retrospective	Japan	LG/RG	1172	TG/DG/PPG/PG
Choi S et al.	2021	Retrospective	Korea	LG/RG/OG	185	TG/DG

TG: Total Gastrectomy; RCT: Randomized Controlled Trial; DG: Distal Gastrectomy; PG: Proximal Gastrectomy; PPG: pylorus-preserving gastrectomy; CG: Completion Gastrectomy.
